# *N*-(4-Bromo­phenyl­sulfon­yl)-2,2,2-tri­methyl­acetamide

**DOI:** 10.1107/S1600536808019375

**Published:** 2008-07-05

**Authors:** B. Thimme Gowda, Sabine Foro, P. G. Nirmala, B. P. Sowmya, Hartmut Fuess

**Affiliations:** a Department of Chemistry, Mangalore University, Mangalagangotri 574 199, Mangalore, India; b Institute of Materials Science, Darmstadt University of Technology, Petersenstrasse 23, D-64287 Darmstadt, Germany

## Abstract

The conformations of the N—H and C=O bonds in the SO_2_—NH—CO—C group of the title compound (N4BPSTMAA), C_11_H_14_BrNO_3_S, are *trans* to each other, similar to what is observed in *N*-(4-chloro­phenyl­sulfon­yl)-2,2,2-trimethyl­acet­amide (N4CPSTMAA) and 2,2,2-trimethyl-*N*-(4-methyl­phenyl­­sulfon­yl)acetamide (N4MPSTMAA). The bond para­meters in N4BPSTMAA are similar to those in N4CPSTMAA, N4MPSTMAA, *N*-aryl-2,2,2-trimethyl­acet­amides and 4-bromo­benzene­sulfonamide. The benzene ring and the SO_2_—NH—CO—C group in N4BPSTMAA form a dihedral angle of 82.8 (1)°, comparable with the values of 82.2 (1)° in N4CPSTMAA and 71.2 (1)° in N4MPSTMAA. N—H⋯O hydrogen bonds form a centrosymmetric ring characterized by an *R*_2_^2^(8) motif.

## Related literature

For related literature, see: Gowda *et al.* (2003 ▶, 2007 ▶, 2008 ▶); Bernstein *et al.* (1995 ▶).
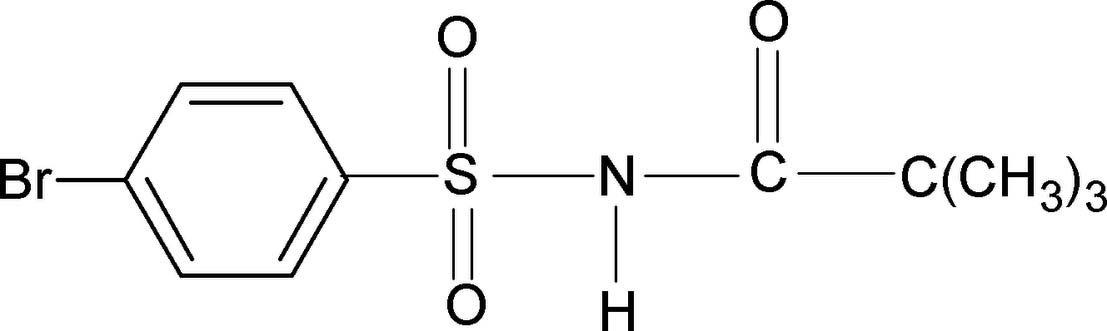


## Experimental

### 

#### Crystal data

C_11_H_14_BrNO_3_S
*M* *_r_* = 320.20
Triclinic, 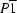

*a* = 6.066 (1) Å
*b* = 10.858 (1) Å
*c* = 11.092 (2) Å
α = 68.19 (1)°
β = 78.66 (2)°
γ = 88.10 (2)°
*V* = 664.40 (17) Å^3^
*Z* = 2
Mo *K*α radiation
μ = 3.25 mm^−1^
*T* = 299 (2) K
0.20 × 0.08 × 0.04 mm

#### Data collection

Oxford Xcalibur diffractometer with Sapphire CCD detector
Absorption correction: multi-scan (*CrysAlis RED*; Oxford Diffraction, 2007 ▶) *T*_min_ = 0.563, *T*_max_ = 0.881
6843 measured reflections
2692 independent reflections
1551 reflections with *I* > 2σ(*I*)
*R*_int_ = 0.033

#### Refinement

*R*[*F* ^2^ > 2σ(*F* ^2^)] = 0.036
*wR*(*F* ^2^) = 0.101
*S* = 0.97
2692 reflections
154 parameters
H-atom parameters constrained
Δρ_max_ = 0.37 e Å^−3^
Δρ_min_ = −0.29 e Å^−3^

### 

Data collection: *CrysAlis CCD* (Oxford Diffraction, 2007 ▶); cell refinement: *CrysAlis RED* (Oxford Diffraction, 2007 ▶); data reduction: *CrysAlis RED*; program(s) used to solve structure: *SHELXS97* (Sheldrick, 2008 ▶); program(s) used to refine structure: *SHELXL97* (Sheldrick, 2008 ▶); molecular graphics: *PLATON* (Spek, 2003 ▶); software used to prepare material for publication: *SHELXL97*.

## Supplementary Material

Crystal structure: contains datablocks I, global. DOI: 10.1107/S1600536808019375/bx2154sup1.cif

Structure factors: contains datablocks I. DOI: 10.1107/S1600536808019375/bx2154Isup2.hkl

Additional supplementary materials:  crystallographic information; 3D view; checkCIF report

## Figures and Tables

**Table 1:** Hydrogen-bond geometry (Å, °)

*D*—H⋯*A*	*D*—H	H⋯*A*	*D*⋯*A*	*D*—H⋯*A*
N1—H1N⋯O2^i^	0.86	2.23	2.982 (3)	146

Symmetry code: (i) 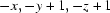.

**Fig. 1.**
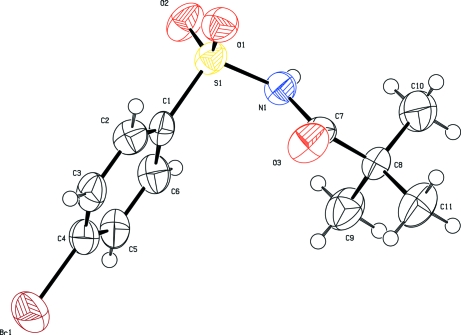
Molecular structure of the title compound, showing the atom labelling scheme. The displacement ellipsoids are drawn at the 50% probability level. H atoms are represented as small spheres of arbitrary radii.

**Fig. 2.**
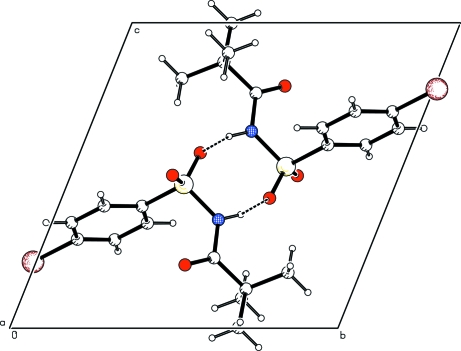
Molecular packing of the title compound with hydrogen bonding shown as dashed lines.

## supplementary crystallographic information

### Comment

In the present work, as part of a study of the substituent effects on the solid state geometries of *N*-(aryl)-sulfonamides and substituted amides, the structure of *N*-(4-bromophenylsulfonyl)-2,2,2-trimethylacetamide (N4BPSTMAA) has been determined (Gowda *et al.*, 2003, 2007, 2008). The conformations of the N—H and C═O bonds of the SO2—NH—CO—C group in N4CPSTMAA are *anti* to each other (Fig. 1), similar to that observed in *N*-(4-chlorophenylsulfonyl)-2,2,2-trimethylacetamide (N4CPSTMAA) and (4-methylphenylsulfonyl)-2,2,2-trimethylacetamide (N4MPSTMAA) (Gowda *et al.*, 2008). The bond parameters in N4BPSTMAA are similar to those in N4CPSTMAA, N4MPSTMAA (Gowda *et al.*, 2008), *N*-(aryl)-2,2,2-trimethylacetamides (Gowda *et al.*, 2007) and 4-bromobenzenesulfonamide (Gowda *et al.*, 2003). The N—H···O hydrogen bonds form a centrosymmetric macro-ring characterized by R_2_^2^(8) motif (Bernstein *et al.*, 1995) ( Table 1, Fig. 2).

### Experimental

The title compound was prepared by refluxing 4-bromobenzenesulfonamide (0.10 mole) with an excess pivalyl chloride (0.20 mole) for about an hour on a water bath. The reaction mixture was cooled and poured into ice cold water. The resulting solid was separated, washed thoroughly with water and dissolved in warm dilute sodium hydrogen carbonate solution. The title compound was precipitated by acidifying the filtered solution with glacial acetic acid. It was filtered, dried and recrystallized from ethanol. The purity of the compound was checked by determining its melting point. It was characterized by recording its infrared and NMR spectra. Single crystals of the title compound used for X-ray diffraction studies were obtained from a slow evaporation of an ethanolic solution.

### Refinement

The H atoms were positioned with idealized geometry using a riding model with C—H = 0.93–0.96 Å, N—H = 0.86 Å, and were refined with isotropic displacement parameters (set to 1.2 times of the *U*_eq_ of the parent atom).

### Crystal data

**Table:**

C_11_H_14_BrNO_3_S	*Z* = 2
*M**_r_* = 320.20	*F*_000_ = 324
Triclinic, *P*1	*D*_x_ = 1.601 Mg m^−^^3^
Hall symbol: -P 1	Mo *K*α radiation λ = 0.71073 Å
*a* = 6.066 (1) Å	Cell parameters from 2537 reflections
*b* = 10.858 (1) Å	θ = 2.3–28.0º
*c* = 11.092 (2) Å	µ = 3.25 mm^−^^1^
α = 68.19 (1)º	*T* = 299 (2) K
β = 78.66 (2)º	Needle, colourless
γ = 88.10 (2)º	0.20 × 0.08 × 0.04 mm
*V* = 664.40 (17) Å^3^	

### Data collection

**Table:**

Oxford Xcalibur diffractometer with Sapphire CCD detector	2692 independent reflections
Radiation source: fine-focus sealed tube	1551 reflections with *I* > 2σ(*I*)
Monochromator: graphite	*R*_int_ = 0.033
*T* = 299(2) K	θ_max_ = 26.4º
Rotation method data acquisition using ω and φ scans	θ_min_ = 2.3º
Absorption correction: multi-scan(CrysAlis RED; Oxford Diffraction, 2007) (Empirical absorption correction using spherical harmonics, implemented in SCALE3 ABSPACK scaling algorithm)	*h* = −7→7
*T*_min_ = 0.563, *T*_max_ = 0.881	*k* = −13→13
6843 measured reflections	*l* = −13→13

### Refinement

**Table:**

Refinement on *F*^2^	Secondary atom site location: difference Fourier map
Least-squares matrix: full	Hydrogen site location: inferred from neighbouring sites
*R*[*F*^2^ > 2σ(*F*^2^)] = 0.036	H-atom parameters constrained
*wR*(*F*^2^) = 0.101	*w* = 1/[σ^2^(*F*_o_^2^) + (0.0526*P*)^2^] where *P* = (*F*_o_^2^ + 2*F*_c_^2^)/3
*S* = 0.97	(Δ/σ)_max_ < 0.001
2692 reflections	Δρ_max_ = 0.37 e Å^−^^3^
154 parameters	Δρ_min_ = −0.29 e Å^−^^3^
Primary atom site location: structure-invariant direct methods	Extinction correction: none

### Special details

**Table:**

Geometry. All e.s.d.'s (except the e.s.d. in the dihedral angle between two l.s. planes) are estimated using the full covariance matrix. The cell e.s.d.'s are taken into account individually in the estimation of e.s.d.'s in distances, angles and torsion angles; correlations between e.s.d.'s in cell parameters are only used when they are defined by crystal symmetry. An approximate (isotropic) treatment of cell e.s.d.'s is used for estimating e.s.d.'s involving l.s. planes.
Refinement. Refinement of *F*^2^ against ALL reflections. The weighted *R*-factor *wR* and goodness of fit *S* are based on *F*^2^, conventional *R*-factors *R* are based on *F*, with *F* set to zero for negative *F*^2^. The threshold expression of *F*^2^ > σ(*F*^2^) is used only for calculating *R*-factors(gt) *etc*. and is not relevant to the choice of reflections for refinement. *R*-factors based on *F*^2^ are statistically about twice as large as those based on *F*, and *R*- factors based on ALL data will be even larger.

### Fractional atomic coordinates and isotropic or equivalent isotropic displacement parameters (Å^2^)

**Table:**

	*x*	*y*	*z*	*U*_iso_*/*U*_eq_	
Br1	−0.10728 (8)	−0.00925 (4)	0.21716 (5)	0.0810 (2)	
S1	0.28409 (14)	0.35519 (8)	0.47093 (8)	0.0451 (2)	
O1	0.4965 (4)	0.3098 (2)	0.5007 (2)	0.0548 (6)	
O2	0.1120 (4)	0.3668 (2)	0.5741 (2)	0.0580 (6)	
O3	0.5763 (4)	0.4572 (2)	0.2073 (2)	0.0615 (7)	
N1	0.3085 (4)	0.5038 (2)	0.3551 (3)	0.0449 (7)	
H1N	0.2309	0.5653	0.3720	0.054*	
C1	0.1807 (5)	0.2548 (3)	0.3993 (3)	0.0399 (8)	
C2	0.2864 (6)	0.1387 (3)	0.4005 (3)	0.0478 (8)	
H2	0.4134	0.1145	0.4378	0.057*	
C3	0.2013 (6)	0.0601 (3)	0.3458 (3)	0.0513 (9)	
H3	0.2703	−0.0176	0.3456	0.062*	
C4	0.0124 (6)	0.0983 (3)	0.2914 (3)	0.0487 (9)	
C5	−0.0926 (6)	0.2124 (3)	0.2896 (4)	0.0521 (9)	
H5	−0.2193	0.2364	0.2520	0.062*	
C6	−0.0080 (5)	0.2915 (3)	0.3443 (4)	0.0506 (9)	
H6	−0.0778	0.3691	0.3440	0.061*	
C7	0.4476 (6)	0.5366 (3)	0.2305 (3)	0.0428 (8)	
C8	0.4154 (5)	0.6724 (3)	0.1284 (3)	0.0451 (8)	
C9	0.1793 (6)	0.6700 (4)	0.0986 (4)	0.0740 (12)	
H9A	0.1665	0.6008	0.0657	0.089*	
H9B	0.1552	0.7540	0.0331	0.089*	
H9C	0.0686	0.6536	0.1782	0.089*	
C10	0.4365 (8)	0.7799 (4)	0.1826 (4)	0.0856 (14)	
H10A	0.3232	0.7639	0.2611	0.103*	
H10B	0.4169	0.8649	0.1172	0.103*	
H10C	0.5828	0.7788	0.2037	0.103*	
C11	0.5872 (7)	0.6969 (4)	0.0018 (4)	0.0759 (12)	
H11A	0.7360	0.6951	0.0197	0.091*	
H11B	0.5660	0.7821	−0.0628	0.091*	
H11C	0.5684	0.6290	−0.0318	0.091*	

### Atomic displacement parameters (Å^2^)

**Table:**

	*U*^11^	*U*^22^	*U*^33^	*U*^12^	*U*^13^	*U*^23^
Br1	0.1133 (4)	0.0498 (3)	0.0888 (4)	−0.0078 (2)	−0.0360 (3)	−0.0269 (2)
S1	0.0570 (6)	0.0369 (5)	0.0389 (5)	0.0063 (4)	−0.0065 (4)	−0.0130 (4)
O1	0.0597 (15)	0.0513 (14)	0.0543 (15)	0.0087 (12)	−0.0183 (13)	−0.0177 (12)
O2	0.0759 (16)	0.0479 (14)	0.0402 (14)	0.0118 (12)	0.0055 (13)	−0.0145 (11)
O3	0.0650 (16)	0.0606 (16)	0.0540 (16)	0.0187 (14)	−0.0034 (13)	−0.0213 (13)
N1	0.0600 (17)	0.0323 (14)	0.0429 (17)	0.0063 (13)	−0.0063 (14)	−0.0169 (13)
C1	0.0397 (18)	0.0325 (17)	0.0390 (18)	−0.0038 (14)	0.0030 (15)	−0.0084 (14)
C2	0.051 (2)	0.0410 (19)	0.051 (2)	0.0136 (16)	−0.0139 (17)	−0.0153 (16)
C3	0.065 (2)	0.0285 (17)	0.054 (2)	0.0035 (16)	−0.0028 (19)	−0.0118 (16)
C4	0.060 (2)	0.0319 (17)	0.052 (2)	0.0014 (16)	−0.0134 (19)	−0.0121 (16)
C5	0.0446 (19)	0.042 (2)	0.059 (2)	−0.0033 (16)	−0.0081 (17)	−0.0081 (17)
C6	0.048 (2)	0.0362 (18)	0.065 (2)	0.0086 (16)	−0.0116 (19)	−0.0167 (17)
C7	0.045 (2)	0.049 (2)	0.0371 (19)	−0.0025 (17)	−0.0069 (16)	−0.0188 (16)
C8	0.048 (2)	0.0432 (19)	0.0356 (19)	−0.0061 (15)	−0.0020 (16)	−0.0076 (15)
C9	0.063 (3)	0.076 (3)	0.065 (3)	0.001 (2)	−0.021 (2)	−0.002 (2)
C10	0.144 (4)	0.041 (2)	0.070 (3)	−0.005 (2)	−0.029 (3)	−0.016 (2)
C11	0.071 (3)	0.077 (3)	0.057 (3)	−0.001 (2)	0.001 (2)	−0.006 (2)

### Geometric parameters (Å, °)

**Table:**

Br1—C4	1.891 (3)	C5—H5	0.9300
S1—O1	1.420 (2)	C6—H6	0.9300
S1—O2	1.430 (2)	C7—C8	1.524 (5)
S1—N1	1.636 (3)	C8—C11	1.514 (5)
S1—C1	1.760 (3)	C8—C10	1.517 (5)
O3—C7	1.208 (4)	C8—C9	1.535 (5)
N1—C7	1.394 (4)	C9—H9A	0.9600
N1—H1N	0.8600	C9—H9B	0.9600
C1—C6	1.380 (4)	C9—H9C	0.9600
C1—C2	1.392 (4)	C10—H10A	0.9600
C2—C3	1.378 (4)	C10—H10B	0.9600
C2—H2	0.9300	C10—H10C	0.9600
C3—C4	1.380 (5)	C11—H11A	0.9600
C3—H3	0.9300	C11—H11B	0.9600
C4—C5	1.369 (4)	C11—H11C	0.9600
C5—C6	1.381 (4)		
			
O1—S1—O2	118.80 (15)	O3—C7—N1	120.2 (3)
O1—S1—N1	111.33 (14)	O3—C7—C8	124.0 (3)
O2—S1—N1	103.71 (14)	N1—C7—C8	115.7 (3)
O1—S1—C1	108.67 (15)	C11—C8—C10	110.9 (3)
O2—S1—C1	109.40 (15)	C11—C8—C7	109.5 (3)
N1—S1—C1	103.87 (14)	C10—C8—C7	110.3 (3)
C7—N1—S1	123.8 (2)	C11—C8—C9	108.6 (3)
C7—N1—H1N	118.1	C10—C8—C9	110.0 (3)
S1—N1—H1N	118.1	C7—C8—C9	107.6 (3)
C6—C1—C2	120.7 (3)	C8—C9—H9A	109.5
C6—C1—S1	119.2 (2)	C8—C9—H9B	109.5
C2—C1—S1	120.1 (2)	H9A—C9—H9B	109.5
C3—C2—C1	119.4 (3)	C8—C9—H9C	109.5
C3—C2—H2	120.3	H9A—C9—H9C	109.5
C1—C2—H2	120.3	H9B—C9—H9C	109.5
C2—C3—C4	119.1 (3)	C8—C10—H10A	109.5
C2—C3—H3	120.5	C8—C10—H10B	109.5
C4—C3—H3	120.5	H10A—C10—H10B	109.5
C5—C4—C3	122.0 (3)	C8—C10—H10C	109.5
C5—C4—Br1	118.6 (3)	H10A—C10—H10C	109.5
C3—C4—Br1	119.5 (2)	H10B—C10—H10C	109.5
C4—C5—C6	119.2 (3)	C8—C11—H11A	109.5
C4—C5—H5	120.4	C8—C11—H11B	109.5
C6—C5—H5	120.4	H11A—C11—H11B	109.5
C1—C6—C5	119.7 (3)	C8—C11—H11C	109.5
C1—C6—H6	120.1	H11A—C11—H11C	109.5
C5—C6—H6	120.1	H11B—C11—H11C	109.5
			
O1—S1—N1—C7	55.9 (3)	C3—C4—C5—C6	−0.3 (5)
O2—S1—N1—C7	−175.2 (2)	Br1—C4—C5—C6	179.6 (3)
C1—S1—N1—C7	−60.9 (3)	C2—C1—C6—C5	−0.1 (5)
O1—S1—C1—C6	−170.6 (3)	S1—C1—C6—C5	−179.0 (3)
O2—S1—C1—C6	58.2 (3)	C4—C5—C6—C1	0.2 (5)
N1—S1—C1—C6	−52.0 (3)	S1—N1—C7—O3	−7.6 (4)
O1—S1—C1—C2	10.5 (3)	S1—N1—C7—C8	169.3 (2)
O2—S1—C1—C2	−120.7 (3)	O3—C7—C8—C11	−6.7 (4)
N1—S1—C1—C2	129.1 (3)	N1—C7—C8—C11	176.5 (3)
C6—C1—C2—C3	0.1 (5)	O3—C7—C8—C10	−129.0 (4)
S1—C1—C2—C3	179.0 (3)	N1—C7—C8—C10	54.2 (4)
C1—C2—C3—C4	−0.2 (5)	O3—C7—C8—C9	111.0 (4)
C2—C3—C4—C5	0.3 (5)	N1—C7—C8—C9	−65.7 (4)
C2—C3—C4—Br1	−179.6 (3)		

### Hydrogen-bond geometry (Å, °)

**Table:**

*D*—H···*A*	*D*—H	H···*A*	*D*···*A*	*D*—H···*A*
N1—H1N···O2^i^	0.86	2.23	2.982 (3)	146

Symmetry codes: (i) −*x*, −*y*+1, −*z*+1.
